# A Study on the Mechanism of Cellulose Nanocrystals to Enhance the Stability of Hydrophobic Phthalocyanine Green in Water and the Functional Characteristics of Colour Pastes

**DOI:** 10.3390/molecules30020327

**Published:** 2025-01-15

**Authors:** Junliang Lu, Jun Xu, Ziyong Zhou, Zhaohui Zhang, Jun Li, Wei Zhang, Kefu Chen

**Affiliations:** 1 Plant Fiber Material Science Research Center, State Key Laboratory of Pulp and Paper Engineering, South China University of Technology, Guangzhou 510640, China; 2 Shandong Sun Paper Industry Joint Stock, Jining 272100, China

**Keywords:** cellulose nanocrystals, phthalocyanine green, binary system, functionalization

## Abstract

Cellulose nanocrystals (CNCs) prepared by sulfuric acid hydrolysis were added to phthalocyanine green colour pastes with a surfactant to improve stability. The particle size, zeta potential, absorbance, and microstructure of the colour pastes were analyzed and characterized. The mechanism of CNCs to enhance the stability of hydrophobic phthalocyanine green in water was investigated. The functionalized application of the colour pastes was explored by mixing colour pastes with coating-forming substances to prepare phthalocyanine green paint. The results show that CNCs could enhance the stability of phthalocyanine green in water and form a binary system with non-ionic surfactants in colour pastes. The system could make phthalocyanine green colour pastes have very good dispersion and stability and mix well with coating-forming substances. The phthalocyanine green paint had excellent performance. As a renewable and naturally degradable biomass resource, CNCs have the potential to be applied for the dispersion and stabilization of hydrophobic pigments by compounding with surfactants.

## 1. Introduction

Phthalocyanine green is a widely used organic pigment which possesses excellent properties, including light fastness, hydrophobicity, weather fastness, and heat stability [1,2]. It has excellent hydrophobic properties and is often mixed with organic solvents to prepare oil-based colour pastes and functional materials. Phthalocyanine green had good dispersibility and a high zeta potential in tetrachloroethylene, so it met the requirements for the preparation of electronic ink and electrophoretic fluid [3,4]. However, the functional application of phthalocyanine green is always limited by organic matter as a dispersing phase. As people pay more attention to the environment and human health, the volatilization of organic compounds in oil-based colour pastes and functional materials has undoubtedly become the main factor hindering the development of phthalocyanine green [5,6,7,8]. Therefore, the preparation of phthalocyanine green water-based colour pastes has important research significance and development potential.

Wetting and dispersing agents are important components of water-based colour pastes and can make solid pigments uniformly dispersed in water. At present, surfactants are the most commonly used wetting and dispersing agents for colour pastes [9,10]. They can be adsorbed on the surface of solid pigments and then quickly wet and disperse the pigments [11]. They provide a theoretical basis for the wetting and dispersion of hydrophobic phthalocyanine green in water. However, evenly dispersed particles will collide and aggregate because of Brownian movement in water and a higher specific surface energy [12,13,14]. They make low-molecular-weight surfactants unable to effectively prevent the aggregation and sedimentation of phthalocyanine green particles. Therefore, these surfactants can not maintain the stability of phthalocyanine green particles well after dispersing the particles. Although some polymer surfactants can ameliorate the problem with their larger steric hindrance effect produced by a high molecular weight, the synthesis steps of the polymer surfactants are complicated and tedious [15,16]. Therefore, it is necessary to find a stabilizer with a simple preparation process and high molecular weight.

Nanocellulose is a green, environmentally friendly, and renewable biomass resource. Cellulose nanofibrils (CNFs) and cellulose nanocrystals (CNCs) are the two most commonly used types of polymer nanocellulose, and their basic structure is cellobiose [17,18]. Therefore, CNFs and CNCs carry a large number of hydroxyl groups and have very good hydrophilic properties and stability in water [19,20,21]. These characteristics give CNFs and CNCs the potential to stabilize solid particles in water. CNFs has good dispersion stability for some solid particles. This is because the surface charge of CNFs is beneficial to improve the dispersion ability of solid particles [22,23]. This is also true for CNCs. Yang et al. [24] used CNCs to disperse and stabilize alkylketene dimers. The results show that CNCs could wrap alkylketene dimer particles in the dispersion process and form a stable suspension. These applications provide some theoretical basis for using nanocellulose as a stabilizer. In terms of preparation technology, the production equipment and steps for creating CNCs are simpler than those for CNFs. CNCs prepared using the acid method are mostly in the form of powder as a commodity and have good dispersion and stability in water [25]. In comparison, the preparation method for CNFs is energy-consuming and time-consuming, and it requires specific mechanical equipment. The equipment can also easily be blocked or damaged during the preparation of CNFs. Their higher maintenance cost and lower production efficiency make CNFs slightly inferior to CNCs regarding preparation technology [26,27]. Therefore, CNCs have greater potential as a stabilizer with a simple preparation process and high molecular weight.

To the best of our knowledge, there are few studies on phthalocyanine green water-based colour pastes and almost no studies on the mechanism of CNCs to enhance the stability of hydrophobic phthalocyanine green in water. In this work, we prepared phthalocyanine green water-based colour pastes using the compound of CNCs colloid and surfactant, and the mechanism of CNCs to enhance the stability of phthalocyanine green in water was investigated by testing the dispersion and stability of the colour pastes. The colour pastes were mixed with coating-forming substances to produce coatings and test functional characteristics. This study provides data and theoretical support for the application of CNCs in the field of hydrophobic pigment and water-based colour pastes.

## 2. Results and Discussion

### 2.1. The Effect of CNCs on the Dispersion and Stability of Phthalocyanine Green in Water

**Figure 1:**
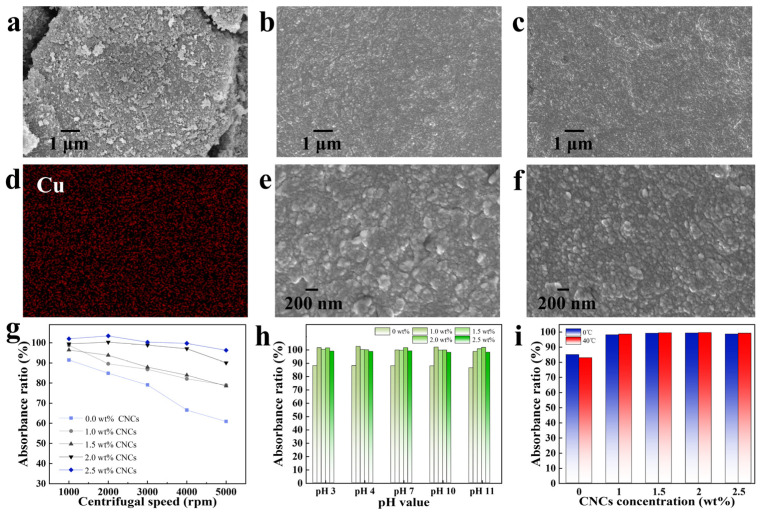
SEM images of phthalocyanine green (**a**), phthalocyanine green colour pastes prepared using 1 (**b**,**e**) and 2.5 wt% (**c**,**f**) CNC colloids. EDS image of phthalocyanine green colour paste prepared using 1 wt% CNC colloids (**d**). Absorbance ratio of phthalocyanine green colour pastes at different rotational speeds (**g**), pH values (**h**), and temperatures (**i**).

The morphology and stability of the phthalocyanine green colour pastes are shown in Figure 1. Compared with phthalocyanine green powder (Figure 1a), the phthalocyanine green particles in colour pastes (Figure 1b,c) had smaller particles that were uniformly dispersed. This visually demonstrates that the phthalocyanine green particles after ultrasonic dispersion did not reaggregate to form the original morphology in the CNCs/op-10 system. The system had a good dispersion effect on the phthalocyanine green particles in water, and these particles were in the nanometer scale (Figure 1e,f). Figure 1d further proves that the phthalocyanine green particles had better dispersion because phthalocyanine green was the only substance containing a copper element in the color pastes (Figure S3).

The stability of the colour pastes was obviously different in the op-10 system and CNCs/op-10 system. Figure 1g shows the r values of the colour pastes after centrifugation for 10 min at different rotational speeds. The ambient temperature of the pastes in the centrifugal process was 25 °C. The phthalocyanine green colour paste in the op-10 system had certain stability at different rotational speeds. This demonstrates that op-10 had the ability to prevent aggregation and sinking of the phthalocyanine green particles in water. However, the ability was weak. The stability of the colour paste was reduced to about 60% at 5000 rpm. The addition of CNCs could effectively improve the stability of the phthalocyanine green colour pastes compared to when CNCs were not added. When the centrifuge speed was at 1000 rpm, the colour pastes added to CNCs all had high stability. With the increase in the centrifugation speed, the colour pastes prepared using 1 and 1.5 wt% CNC colloids showed a downward trend, but their stability at the same speed was still significantly higher than that of the colour paste containing only op-10. The downward trend was not obvious in the colour pastes prepared using 2 and 2.5 wt% CNC colloids. The stability of phthalocyanine green colour pastes in the CNCs/op-10 system was significantly higher than that in the op-10 system. It was proven that CNCs could effectively enhance the stability of hydrophobic phthalocyanine green in water, and this enhancement effect was enhanced with the increase in the CNC solid content.

Furthermore, the addition of CNCs could also maintain high stability of the colour pastes in different pH values and temperature environments. The pH values were changed by adding HCl and NaOH. Figure 1h,i show the stability of the colour pastes in different environments for 30 days. Among them, the temperature of the samples in Figure 1h was 25 °C. The r values of the colour pastes in op-10 system were between 80 and 90%, but the r values of the colour pastes in CNCs/op-10 system were close to 100%. These colour pastes continued to be placed in the same environment, and it was surprisingly found that the stability of the colour pastes in the CNCs/op-10 system was still close to 100% after 180 days (Figure S4). This further proves that the stability of the phthalocyanine green colour pastes in the CNCs/op-10 system was significantly higher than those in the op-10 system, and CNCs could effectively improve the stability of phthalocyanine green in water. This ability of CNCs was almost unaffected by pH and temperature. Therefore, the CNCs/op-10 system had an important effect on the dispersibility and stability of phthalocyanine green in water. The phthalocyanine green particles in water could have good dispersion and stability in the CNCs/op-10 system. The color pastes with a higher CNC solid content could better maintain stability at different rotational speeds, pH values, and temperatures.

### 2.2. An Analysis of the Ability of CNCs to Enhance the Stability of Hydrophobic Phthalocyanine Green in Water

The phthalocyanine green colour pastes in the CNCs/op-10 system showed good dispersibility and stability, as shown in Figure 1. In general, solid particles were dispersed and stably suspended in the liquid phase by charge repulsion and steric hindrance [28,29,30]. In addition, some solid particles could form chemical bonds with substances stably dispersed in the liquid phase. The particles could also maintain good dispersion and stability in the liquid phase [31]. As a solid pigment, the dispersion and stabilization mechanism of phthalocyanine green in the op-10 system and CNCs/op-10 system should follow some of the above mechanism.

**Figure 2:**
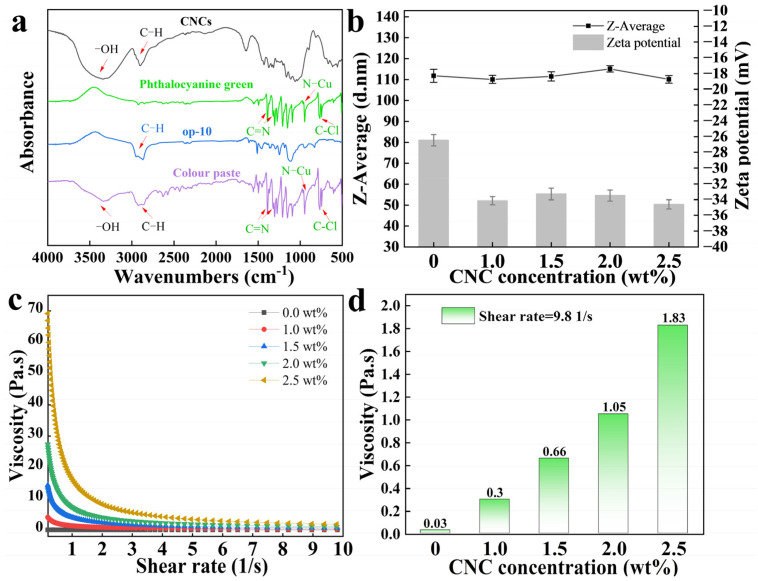
(**a**) IR spectra of CNCs, phthalocyanine green, op-10, and phthalocyanine green colour paste with CNCs; (**b**) size and zeta potential of colour pastes; (**c**,**d**) shear viscosity of colour pastes.

Figure 2a shows the infrared test results of the CNCs, phthalocyanine green, op-10, and phthalocyanine green colour paste. Through the comparison of the four curves, it can be seen that the curve of the colour pastes not only contains all the characteristic peaks of CNCs, phthalocyanine green, and op-10, but also has no new characteristic peaks. The colour pastes and CNCs curves all show a wide and strong characteristic peak near 3400 cm^−1^, which is the stretching vibration peak on the hydroxyl group, and bending vibration peaks appear near 1638 cm^−1^. In the CNCs, op-10, and colour paste curves, the stretching vibration peaks of the carbon–hydrogen bond appear near 2910 cm^−1^ and 2850 cm^−1^. When rare in number and insufficient in strength, the carbon–hydrogen bond often presents as a single peak at 2910 cm^−1^. The characteristic peak position of the colour paste after 1500 cm^−1^ is mainly the same as that of phthalocyanine green. The C=N stretching vibration in the isoindazole structure appears at 1390 cm^−1^ and 1326 cm^−1^. The characteristic peaks at 948 cm^−1^ and around 600–800 cm^−1^ represent the existences of N-Cu and C-Cl, respectively [4]. Moreover, the characteristic peaks of op-10 after 1500 cm^−1^ are also the same as the colour paste curve. This suggests that no new chemical bonds were formed during the preparation of the colour pastes. Therefore, the preparation of phthalocyanine green colour pastes was a purely physical reaction.

Figure 2b shows the results of the zeta potential and Z-average particle size of the phthalocyanine green colour pastes. The zeta potentials of the colour pastes are all negative. This proves that the phthalocyanine green particles carried some negative charges and repelled each other in the op-10 system and CNCs/op-10 system. There was a charge repulsion effect in these colour pastes, meaning they could maintain the stability of phthalocyanine green particles in water. Meanwhile, the colour pastes with the addition of CNCs had higher absolute values of the zeta potential than the colour paste without CNCs. Therefore, adding CNCs to the colour pastes was conducive to improving the charge-carrying capacity of the phthalocyanine green particles and enhancing the stability of phthalocyanine green in water. Nevertheless, the absolute values of the zeta potential did not change significantly with the increase in the CNC solid content, and the CNCs could not significantly affect the particle size of the colour paste. This proves that the charge amount of phthalocyanine green particles was almost unaffected by the content of CNCs in the colour pastes, and the particle size was mainly affected by op-10 rather than CNCs. The similar particle size made these particles have similar specific surface areas and adsorption capacity for negative charges. This could be the reason why the zeta potential was almost not affected by the CNC solid content in the colour pastes.

Figure 2c,d show the shear viscosity of the colour pastes. The colour pastes prepared by CNC colloids with different solid contents had different viscosities at the same shear rate, and the colour pastes with a high solid content of CNCs had higher viscosities. The viscosities of the colour pastes with CNCs all gradually decreased with the increase in the shear rate, but the viscosity of the colour paste without CNCs was almost unchanged and significantly lower than the other colour pastes. The phenomenon of fluid thinning due to shearing was similar to CNC colloids [32]. This proves that CNCs could still form a network structure in the colour pastes. The structure could produce a steric hindrance effect on the phthalocyanine green particles to enhance the stability of hydrophobic phthalocyanine green in water and significantly improve the viscosity of the colour pastes. In summary, phthalocyanine green particles were stably suspended in the aqueous phase by charge repulsion and steric hindrance, and the preparation of colour pastes caused a physical reaction. Using CNCs in the colour pastes could significantly improve the amount of charge on the particles and the steric hindrance of the liquid phase to the particles. Therefore, the phthalocyanine green colour pastes with CNCs had higher stability. CNCs could improve the stability of hydrophobic phthalocyanine green in water.

### 2.3. The Mechanism of CNCs to Enhance the Stability of Hydrophobic Phthalocyanine Green in Water

**Figure 3:**
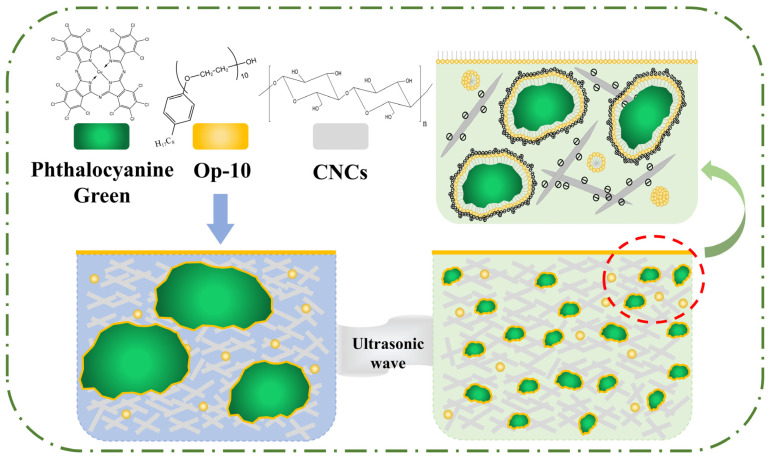
Mechanism diagram of ability of CNCs to enhance stability of hydrophobic phthalocyanine green in water.

Figure 3 shows the mechanism of CNCs to enhance the stability of hydrophobic phthalocyanine green in water. When only phthalocyanine green was added to the water, the strong hydrophobicity of phthalocyanine green caused aggregation, and phthalocyanine green could not be wetted by the aqueous phase. With the addition of op-10, the surface of the phthalocyanine green particles was gradually wrapped by op-10. Together, the phthalocyanine green particles and op-10 formed core–shell structures. The particles as cores were wrapped in a molecular shell of op-10. The op-10 molecule had hydrophilic and hydrophobic groups. The hydrophobic groups were adsorbed on the surface of the particles, and the hydrophilic group was in contact with the aqueous phase due to the properties of the surfactants [33,34] (Figure S5). Therefore, op-10 could achieve phthalocyanine green wetting with water. Phthalocyanine green, op-10, and water formed a simple colour paste system.

When fixed-energy ultrasonic waves were injected into the system, the phthalocyanine green particles were broken into smaller particles by the ultrasonic waves. The small particles could reconstitute smaller core–shell structures with op-10 and disperse in the water. These core–shell structures had a tendency to aggregate with each other and sink due to Brownian motion and gravity [13]. This was the main factor that destroyed the stability of the colour paste. Meanwhile, they carried a negative charge in the water (Figure 2b). This made a charge repulsion effect exist between the core–shell structures and was conducive to maintaining the stability of the colour paste. Moreover, the molecular shell around the phthalocyanine green particles could also prevent the aggregation and sinking of the particles. Therefore, the phthalocyanine green colour paste was stable in the aqueous phase with the addition of op-10. However, the stability was weak when relying solely on op-10. The colour paste in which only op-10 was added could not maintain stability in complex external environments for a long time (Figure 1g–i). When CNCs were added to the colour paste system, the stability of the colour paste was significantly improved. This was because CNCs have a higher molecular weight and can significantly enhance the zeta potential of the colour paste. CNCs could interweave with each other to form a network structure in water due to hydroxyl groups on their surface. The structure made a large number of CNCs exist around each phthalocyanine green particle, and the particles were be hindered by the strong steric hindrance effect of CNCs during the process of aggregation and subsidence. In addition, there was charge repulsion between the particles and CNCs due to the negative charge carried by the CNCs [35]. The steric hindrance effect and charge repulsion of CNCs could significantly enhance the stability of the colour paste. Therefore, CNCs could effectively enhance the stability of hydrophobic phthalocyanine green in water.

### 2.4. Functional Characteristics of Phthalocyanine Green Colour Pastes

Simple water-based paint was prepared by mixing phthalocyanine green colour pastes prepared using 0, 1, 1.5, 2, and 2.5 wt% CNC colloids with acrylic emulsion, and the paint was applied on wood, polypropylene, stainless steel, and tinplate to prepare the coatings. The mass ratio of colour pastes and acrylic emulsion was 1:2. The adhesion, water resistance, and glossiness of the phthalocyanine green coatings were tested. Table 1 shows the adhesion of coatings on the wood, polypropylene, and stainless steel. The adhesion level of the coatings was 0 or 1 on these materials. According to the standard of ISO 2409:2020 (en) “Paints and varnishes-Cross-cut test”, coatings with an adhesion level of 0 or 1 level had good adhesion [36]. Therefore, phthalocyanine green paint had good adhesion to wood, polypropylene, and stainless steel. The adhesion level of the coatings on the same material was the same, so the solid content of CNCs had no significant effect on the adhesion of the coatings.

**Figure 4:**
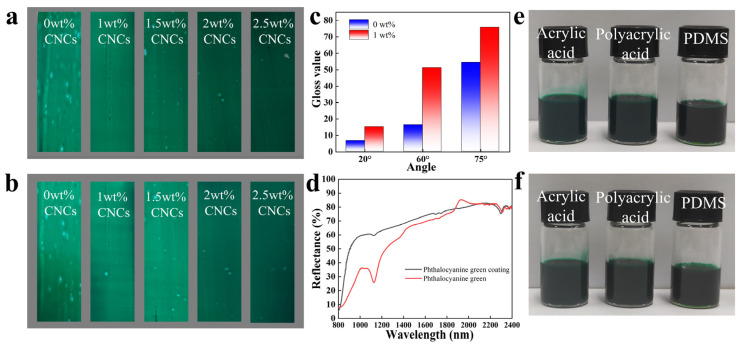
The coatings after being cooked for 0 h (**a**) and 3 h (**b**). The glossiness of the coatings (**c**). The near-infrared reflectance of the phthalocyanine green powder and coating (**d**). The paint after 0 days (**e**) and 5 days (**f**).

The coatings on the tinplate were tested for water resistance, glossiness, and near-infrared reflectance. These coatings did not peel, blister, or lose light after cooking for 3 h in the water resistance test (Table 2, Figure 4a,b). This proves that phthalocyanine green paint had good water resistance. Among them, the coatings containing colour pastes prepared using 0 and 1 wt% CNC colloids were tested for glossiness (Figure 4c). The coating with CNCs had higher glossiness values. This proves that CNCs could significantly improve the glossiness of the paint and make the coatings brighter. The near-infrared reflection test results prove that phthalocyanine green had near-infrared reflection ability (Figure 4d). The phthalocyanine green powder had high reflectance at a range of 1800–2500 nm, and the coating containing colour paste prepared using 0 wt% CNC colloids had a better reflection effect in the entire near-infrared band. Therefore, phthalocyanine green colour pastes could be used as near-infrared material and have the potential to be used as near-infrared reflective coatings.

Moreover, the phthalocyanine green colour paste prepared using 1 wt% CNC colloids was mixed with water-based (polyacrylic acid) and oil-based (PDMS) coating-forming substances to expand the application range, respectively. This phthalocyanine green paint had no noticeable delamination after 5 days (Figure 4e,f). This proves that CNCs did not affect the mixing effect of phthalocyanine green colour pastes and coating-forming substances, and the colour pastes had good compatibility and stability with water-based emulsion and water-based and oil-based coating-forming substances.

**Figure 5:**
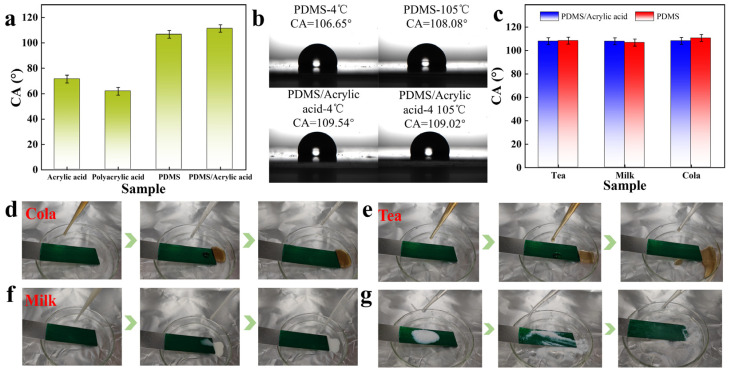
The contact angles of the phthalocyanine green coatings (**a**–**c**). Images of cola, tea, and milk easily rolling off the surfaces of the coatings (**d**–**f**). The self-cleaning properties of the phthalocyanine green coatings (**g**).

Among them, the hydrophilicity and hydrophobicity of the paint were mainly determined by coating-forming substances. The paint with water-based material as a coating-forming substance had smaller values of the contact angle with water and better hydrophilicity. In contrast, the paint made by mixing PDMS and a colour paste had better hydrophobicity due to the hydrophobic properties of PDMS. This paint was made into a coating and tested for its contact angle with water, as shown in Figure 5a. The contact angle of the coating containing PDMS was greater than 100°, and a similar value of contact angle could still be obtained when PDMS was applied to the surface of the coating containing an acrylic emulsion. The colour pastes did not affect the hydrophobicity of PDMS. Better hydrophobicity could maintain the cleaning of the coating surface and was conducive to maintaining the function of the phthalocyanine green colour pastes. Figure 5b shows the hydrophobic stability of coatings at different temperatures. The coatings still had good hydrophobicity at lower and higher temperatures. Additionally, the hydrophobicity of these coatings was not just limited to water. Tea, milk, and cola still had large contact angle values on the surfaces of the coatings (Figure 5c). Therefore, PDMS could make phthalocyanine green colour pastes and paint have good hydrophobicity, and the colour pastes almost did not affect the self-cleaning properties of PDMS. These liquids also could not adhere to the coating containing PDMS. They quickly slid off the coating while the coating remained tilted, and the coating remained clean (Figure 5d–f). The self-cleaning property of the coating was tested by simulating dust with hydrophobic silica. The hydrophobic silica on the surface of the coating could be easily washed away by water (Figure 5g). This proves that the coating containing PDMS was self-cleaning and could maintain the cleaning of the coating surface. In summary, phthalocyanine green colour pastes had good functional characteristics. These characteristics came from phthalocyanine green and CNCs. The colour pastes had good compatibility with commonly used coating-forming substances and almost did not affect the properties of the substances. Phthalocyanine green colour pastes could be used as good functional material in the field of coatings.

## 3. Materials and Methods

### 3.1. Chemicals and Materials

Cellulose nanocrystals were obtained from ScienceK Co., Ltd. (Huzhou, China), which were prepared by sulfuric acid hydrolysis and spray drying, and the details are shown in the Supporting Information (Figures S1 and S2). Phthalocyanine green was purchased from Kedi New Material Technology Co., Ltd. (Guangzhou, China). Op-10 emulsifier and polyacrylic acid were purchased from Maclin Biochemical Technology Co., Ltd. (Shanghai, China). Polydimethylsiloxane (PDMS) was obtained from Dow Corning (Midland, MI, USA). Acrylic emulsion was provided by the Jitian Co., Ltd. (Shenzhen, China). Hydrochloric acid and sodium hydroxide were provided by Sinopharm Chemical Reagent Co., Ltd. (Shanghai, China). Tea, milk, and cola were provided by the local supermarket. Distilled water was prepared in the laboratory.

### 3.2. Preparation of CNCs Colloid

Cellulose nanocrystal powder was prepared using the sulfuric acid method. Powder with different absolute dry weights was mixed with water, magnetically stirred for 10 min, and then ultrasonically dispersed for 10 min using a cell crusher (VCX800, Sonics, Newtown, CT, USA). CNC colloids with mass percentages of 1.0, 1.5, 2.0, and 2.5 wt% were prepared.

### 3.3. Preparation of Phthalocyanine Green Colour Pastes and Paint

A certain amount of dried phthalocyanine green powder was weighed and placed into a beaker. The CNC colloid with op-10 (CNCs/op-10 system) or distilled water with op-10 (op-10 system) was added into the beaker and mixed with the powder. The phthalocyanine green colour pastes in CNCs/op-10 system were prepared by ultrasonically dispersing the mixture. The mass ratio of op-10, phthalocyanine green, and CNC colloid was 1:10:30. The total input energy of the cell crusher was 10,000 J. The phthalocyanine green colour pastes in the op-10 system were prepared under the same conditions as the control group. The mass ratio of op-10, phthalocyanine green, and distilled water was 1:10:30. Then, phthalocyanine green paint was prepared by mixing these phthalocyanine green colour pastes with coating-forming substances according to a mass ratio of 1:2. The coating-forming substances were acrylic emulsion, polyacrylic acid, and PDMS, which were separately mixed with the colour pastes.

### 3.4. Morphology of Phthalocyanine Green Colour Pastes

The phthalocyanine green powder and phthalocyanine green colour pastes were prepared into thin and uniform samples. The samples were coated with conductive adhesive and treated with gold spray. The morphology of the samples was observed by SEM (Merlin, Carl Zeiss AG, Oberkochen, Germany) under different multiples. The samples were analyzed using an energy-dispersive spectrum analyzer (Oxford Instruments, Oxford, UK). In addition, the phthalocyanine green powder, CNCs, op-10, and colour pastes were dried and mixed with potassium bromide powder to analyze the mixture using Fourier-transform infrared spectroscopy (FT/IR-4700, JASCO Corporation, Tokyo, Japan) in the range of 500–4000 cm^−1^.

### 3.5. Measurement of Dispersibility and Stability of Phthalocyanine Green Colour Pastes

#### 3.5.1. Particle Size and Zeta Potential

A small amount of phthalocyanine green colour pastes were diluted 10,000 times by water to obtain transparent suspension. The Z-average particle size and zeta potential of transparent suspension were tested using a particle size analyzer (ZetasizerNano-ZS90, Malvern Instruments, Malvern, UK). During the test, the system temperature was 25 °C and the equilibrium time was 60 s.

#### 3.5.2. Viscosity

The viscosity of the phthalocyanine green colour pastes was measured using a rheometer (DHR 1, TA Instruments, New Castle, DE, USA) with a parallel plate (diameter 25 mm) and gap fixed at 1000 μm. The viscosity at different shear rates in the range of 0.01–10 s^−1^ was measured at 25 °C within 3 min.

#### 3.5.3. Absorbance

A small amount of newly prepared phthalocyanine green colour pastes were diluted 10,000 times by water to obtain a transparent solution. The absorbance, *A*_0_, of the transparent solution was measured using an ultraviolet spectrophotometer (UV2600, Shimadzu Company, Kyoto, Japan) at a wavelength of 650 nm. The phthalocyanine green colour pastes were centrifuged at different speeds for 10 min or stood for a long time in different environments, and then we took a small amount of upper liquid at the same test wavelength and carried out multiple dilution to determine the absorbance, *A*_1_. The stability of phthalocyanine green colour pastes was proven by the ratio of absorbance (*r*). The larger the *r* value, the better the stability of the colour pastes. The *r* value was calculated using Equation (1):

(1) $$r=\frac{A_{1}}{A_{0}}\times 100\%$$

### 3.6. Functional Measurement of Phthalocyanine Green Colour Pastes

The phthalocyanine green colour pastes were mixed with 30 wt% acrylic emulsion according to a mass ratio of 1:2 to make a simple paint. Subsequently, the phthalocyanine green paint was evenly coated on the wood, polypropylene, stainless steel, and tinplate to prepare the coatings. The adhesion of the coatings coated on the wood, polypropylene, and stainless steel was tested with reference to ISO 2409:2020 (en) “Paints and varnishes-Cross-cut test” [36]. The specific operation was as follows: six parallel cuts were introduced in the coating, and another six cuts were introduced perpendicular to the first cuts. Any loose paint particles were removed. The cut area was examined visually and compared to a six-step classification.

The coatings coated on the tinplate were tested for light reflectance using an ultraviolet-visible near-infrared (UV-vis-NIR) spectrophotometer (Lambda 950, PerkinElmer, Shelton, CT, USA) with a wavelength in the range of 800–2400 nm and tested for glossiness using a Novo-Gloss tester (Novo-Gloss, Rhopoint, East Sussex, UK). Then, the coatings were cooked in boiling water for 3 h to test water resistance. Moreover, the phthalocyanine green colour pastes were also mixed with polyacrylic acid and PDMS to expand the application range. The paint mixed with PDMS was also tested for static contact angle (CA) using a contact angle metre (ZJ-7000, Zhijia equipment, Shenzhen, China) and for self-cleaning ability due to the hydrophobicity of PDMS.

## 4. Conclusions

In this study, the dispersion and stabilization effect of the CNCs/op-10 system on phthalocyanine green were investigated. It was found that CNCs and op-10 had good dispersion and stabilization effects on phthalocyanine green in an aqueous phase. The prepared phthalocyanine green colour pastes had very strong resistance to centrifugation, pH, high temperatures, and low temperatures. In the system, CNCs could significantly enhance the stability of phthalocyanine green in water, and op-10 mainly played a role in the wetting of phthalocyanine green. The mechanism of CNCs to enhance the stability of hydrophobic phthalocyanine green in water was analyzed. Steric hindrance and charge repulsion were the main factors of the enhancement effect. In addition, the colour pastes had good compatibility and stability with water-based emulsion and with water-based and oil-based coating-forming substances. As a renewable and naturally degradable biomass resource, CNCs have potential to be combined with op-10 to become a new dispersant and stabilizer for the dispersion and stabilization of phthalocyanine green in an aqueous phase.

## Figures and Tables

**Table 1:** The adhesion of the phthalocyanine green coatings.

Sample	Wood	Polypropylene	Stainless Steel
0.0 wt% CNCs	0	1	0
1.0 wt% CNCs	0	1	0
1.5 wt% CNCs	0	1	0
2.0 wt% CNCs	0	1	0
2.5 wt% CNCs	0	1	0

**Table 2:** The water resistance of the phthalocyanine green coatings.

Sample	Peeling	Blistering	Loss of Light
0.0 wt% CNCs	NO	NO	NO
1.0 wt% CNCs	NO	NO	NO
1.5 wt% CNCs	NO	NO	NO
2.0 wt% CNCs	NO	NO	NO
2.5 wt% CNCs	NO	NO	NO

## Data Availability

Data are contained within the article and Supplementary Materials.

## Supplementary Materials

The following supporting information can be downloaded at: https://www.mdpi.com/article/10.3390/molecules30020327/s1. Figure S1. (a) SEM image of CNCs; (b) Zeta potential of CNCs. Figure S2. (a) The hydrodynamic diameter of CNCs; (b) TEM image of CNCs; (c) AFM image of CNCs; (d) SEM image of CNCs. Figure S3. Structural formula for phthalocyanine green, op-10 and CNCs. Figure S4. The absorbance ratio of phthalocyanine green colour pastes at different pH values and temperatures after 180 days. Figure S5. Adsorption mechanism diagram of phthalocyanine green by op-10 in water.
