# Supplementary material for: A Study on the Mechanism of Cellulose Nanocrystals to Enhance the Stability of Hydrophobic Phthalocyanine Green in Water and the Functional Characteristics of Colour Pastes
Source: Molecules. 2025 Jan 15;30(2):327. doi: 10.3390/molecules30020327 (PMC11767406; doi:10.3390/molecules30020327)
Supplement: Supplementary file 1 [file molecules-30-00327-s001.zip › molecules-3337432-supplementary.pdf]

Supporting information

A Study on the Mechanism of Cellulose Nanocrystals to Enhance the Stability of Hydrophobic Phthalocyanine Green in Water and the Functional Characteristics of Colour Pastes

Junliang Lu[1], Jun Xu[1, 2] *, Ziyong Zhou[1], Zhaohui Zhang[1], Jun Li[1], Wei Zhang[2], Kefu Chen[1]

[1]Plant Fiber Material Science Research Center, State Key Laboratory of Pulp and Paper Engineering, South China University of Technology, Guangzhou, 510640, China.

[2]Shandong Sun Paper Industry Joint Stock, Jining 272100, China.

*Corresponding author: Jun Xu

E-mail address: xujun@scut.edu.cn

Characterization of CNCs:

Cellulose nanocrystals (CNCs) were prepared by sulfuric acid and spray drying. The CNCs carried negative charges on the surface, and could be uniformly dispersed in water to form colloid. The zeta potential values of the CNCs colloid with different solid content were all higher than 40, which was consistent with the stability index of the colloidal materials. The dimension of CNCs was assessed by scanning electron microscopy (SEM, Merlin compact, Zeiss, Germany). The zeta potential of CNCs was tested by potential analyzer (ZetasizerNano-ZS90, Malvern Instruments, UK).

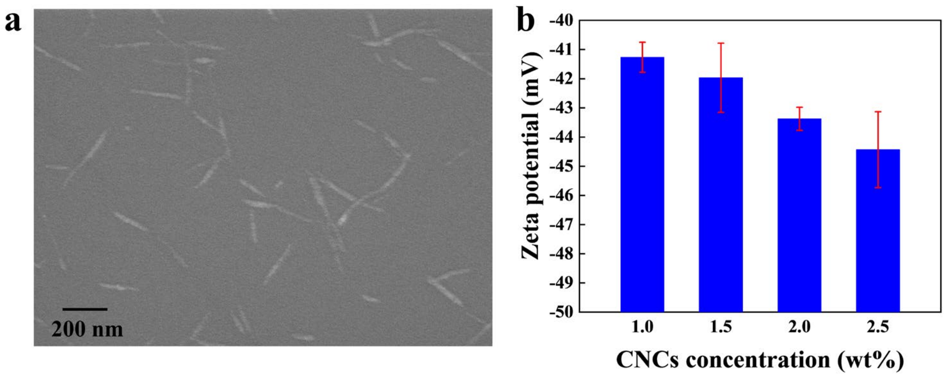

Fig.S1. (a) SEM image of CNCs; (b) Zeta potential of CNCs.

In addition, the supplier of CNCs (ScienceK Co., Ltd., Huzhou, China) also provided product information, as shown in Fig.S2.

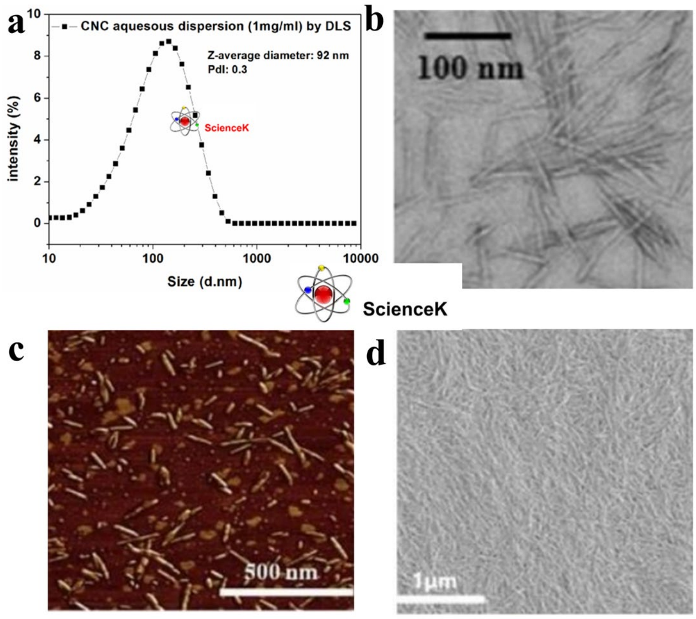

Fig.S2. (a) The hydrodynamic diameter of CNCs; (b) TEM image of CNCs; (c) AFM image of CNCs; (d) SEM image of CNCs.

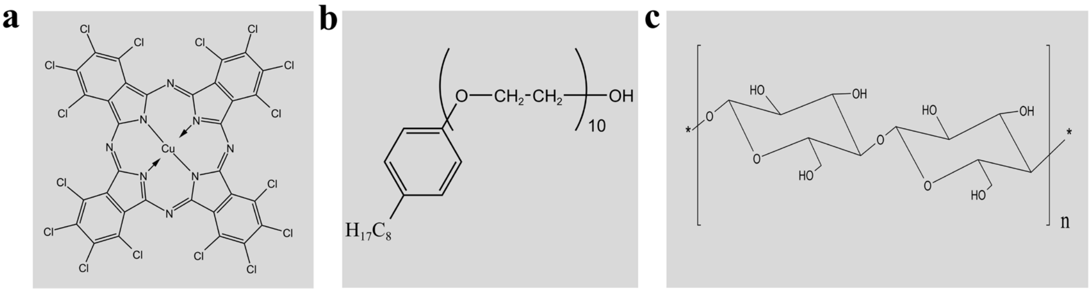

Phthalocyanine Green **Op-10** **CNCs**

Fig.S3. Structural formula for phthalocyanine green, op-10 and CNCs.

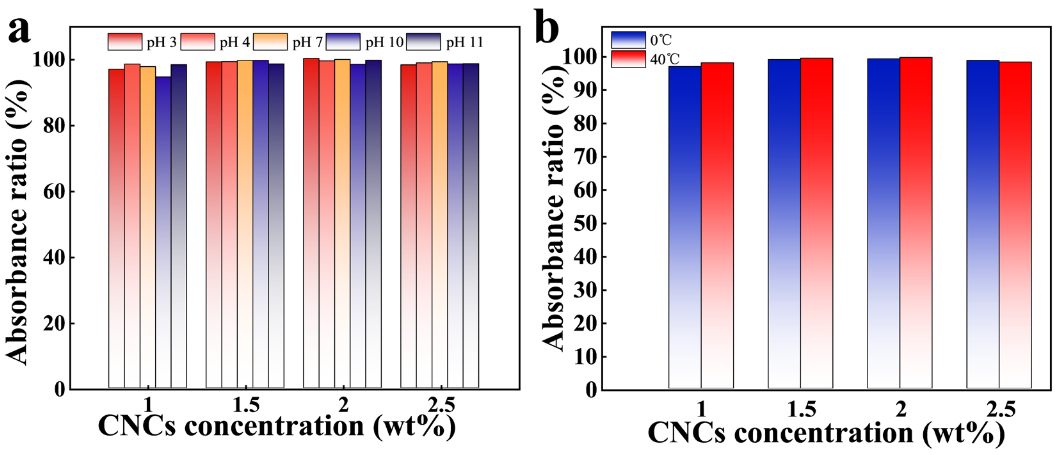

Fig. S4. The absorbance ratio of phthalocyanine green colour pastes at different pH values and temperatures after 180 days.

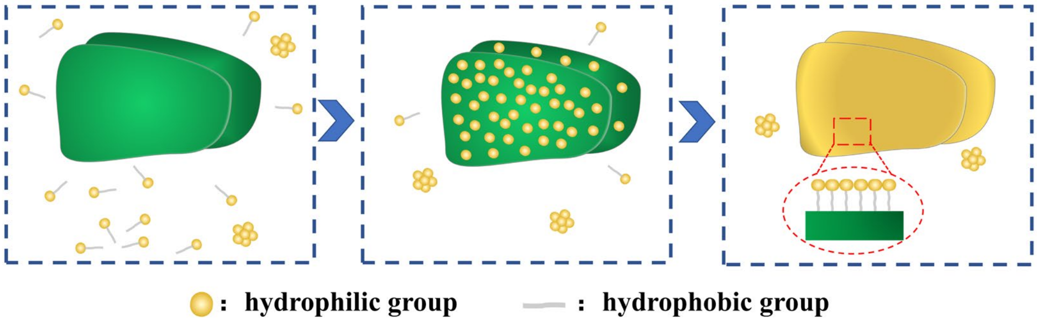

Fig. S5. Adsorption mechanism diagram of phthalocyanine green by op-10 in water.
